# In This Issue: Issue 116, Volume 11, November 2025

**DOI:** 10.1111/cas.70238

**Published:** 2025-11-03

**Authors:** Hiroyuki Seimiya

**Affiliations:** ^1^ Japanese Foundation for Cancer Research, Cancer Chemotherapy Center Tokyo Japan

## The Impact of Polyploid Giant Cancer Cells: The Root of Stress Resilience

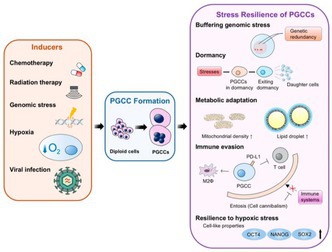

Sometimes cancer cells grow so large and abnormal that they no longer resemble typical cells. They may contain massive, or even multiple, nuclei packed with extra DNA. These unusual cells are called Polyploid Giant Cancer Cells, or PGCCs. While not widely known, scientists are increasingly realizing that they may be among the most dangerous cancer cell types.

PGCCs tend to appear when cancer is under attack, such as after chemotherapy, radiation, or other harsh conditions that would normally kill most cells. Instead of dying, these giant cells survive, adapt, and may even help the tumor regrow. PGCCs have been found in breast, ovarian, liver, prostate, and other cancers, and are often linked to poor outcomes. Importantly, they can also arise before any treatment has been given.

In this review, Ogawa et al. explain why PGCCs are especially concerning. Their extra DNA can act as a backup system against damage, they can “hibernate” in a dormant state until conditions improve, and they adapt to survive low oxygen, poor nutrition, and toxic drugs. PGCCs may also evade the immune system. They do this by releasing signaling molecules, displaying surface proteins that switch off immune cells, or influencing nearby immune cells to support tumor growth. These strategies make them even harder to eliminate.

Despite their importance, scientists still lack reliable markers to identify PGCCs. Current research is exploring new imaging and AI‐based approaches, as well as potential therapies—including drugs such as zoledronic acid and mifepristone—that may one day help target these resilient cells and improve cancer treatment outcomes.

https://onlinelibrary.wiley.com/doi/full/10.1111/cas.70191

## Mitochondrial DNA‐Mediated Intercellular Communication via Extracellular Vesicles Under Hypoxic Stress to Drive HCC Progression

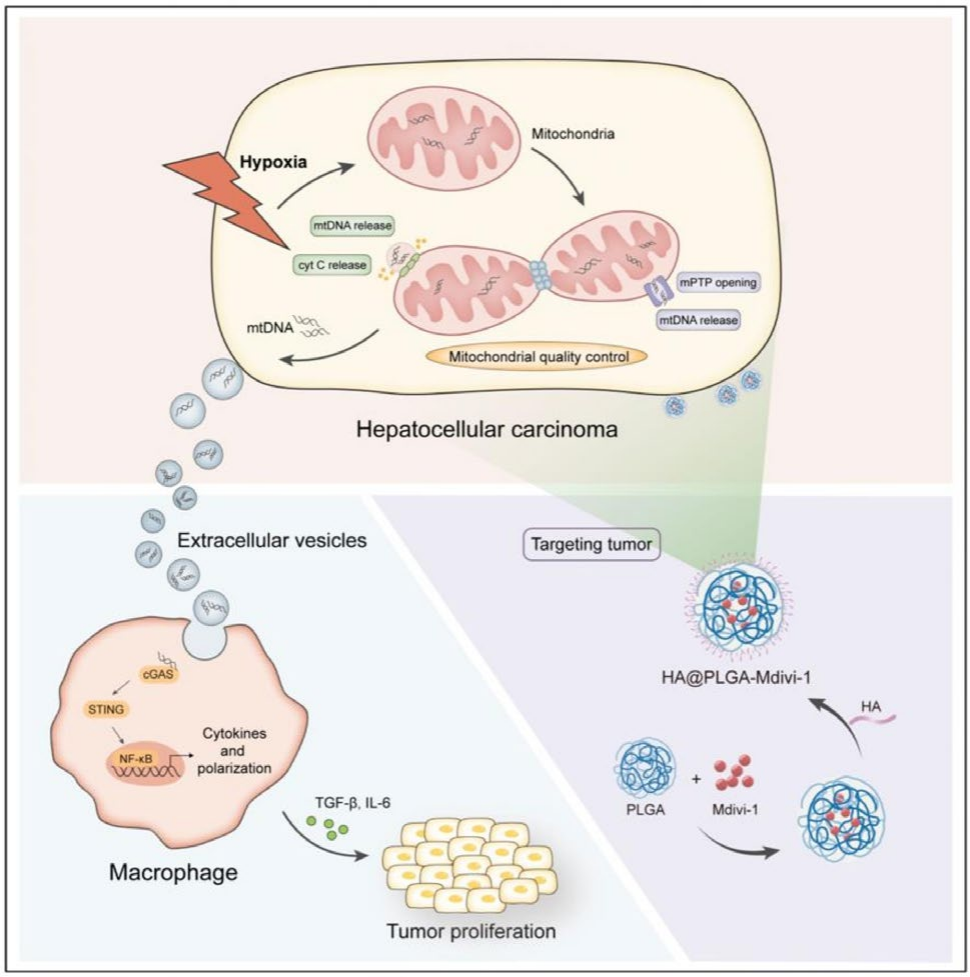

Hepatocellular carcinoma (HCC) is a type of cancer characterized by the abnormal proliferation of liver cells. The highly lethal nature of HCC is due to its resistance to treatment. Efforts are ongoing to develop new strategies to improve cancer treatment outcomes. One such strategy is immunotherapy, which works by activating the body's immune system to kill cancer cells. For immunotherapy to be effective, various immunity‐related factors and cells must be activated in the cancer “microenvironment.” However, the low oxygen conditions in HCC (hypoxia) prevent the activation of anti‐cancer immune responses, which can be mainly attributed to the presence of a specific group of immune cells called tumor‐associated macrophages (TAMs).

Cancer cells employ various strategies to escape the host immune response. One such strategy involves passing signals to immune cells through extracellular vesicles (EVs), which are spherical structures that can carry signaling molecules. Hypoxia can also damage the mitochondria, which are cellular organelles that generate energy using oxygen. Mitochondria harbor their own DNA, referred to as mitochondrial DNA (mtDNA). However, the role of mtDNA in HCC development has remained unclear.

This study revealed that hypoxia promotes the release of mtDNA from the mitochondria in HCC cells. The mtDNA is subsequently packed and transported outside the cells through EVs. Once outside the cells, mtDNA in EVs signals some immune cells to transform into M2 macrophages, which are similar to TAMs. EVs derived from HCC cells cultured under hypoxic conditions could promote the secretion of transforming growth factor‐beta (a cell division‐promoting protein) and consequently increase HCC cell proliferation. Our findings indicate that mtDNA released from HCC cells via EVs in response to hypoxia can not only prevent immune responses against HCC but also enhance HCC growth.

Given the importance of EVs‐mtDNA in HCC progression, it can also be targeted for developing new therapeutic modalities. The researchers prepared a nanoparticle formulation containing an inhibitor (Mdivi‐1) of a mitochondrial division–related protein (Drp1). Nanoparticle formulations enable effective and precise drug delivery. The nanoparticles were effectively taken up by tumor cells and inhibited the growth of tumors in animals with HCC. This study has important implications for developing novel treatments for HCC.

https://onlinelibrary.wiley.com/doi/full/10.1111/cas.70172

## SOX10 Regulates Melanoma Metastasis Through the IRF1‐ITGA3/EphA2‐FAK Pathway

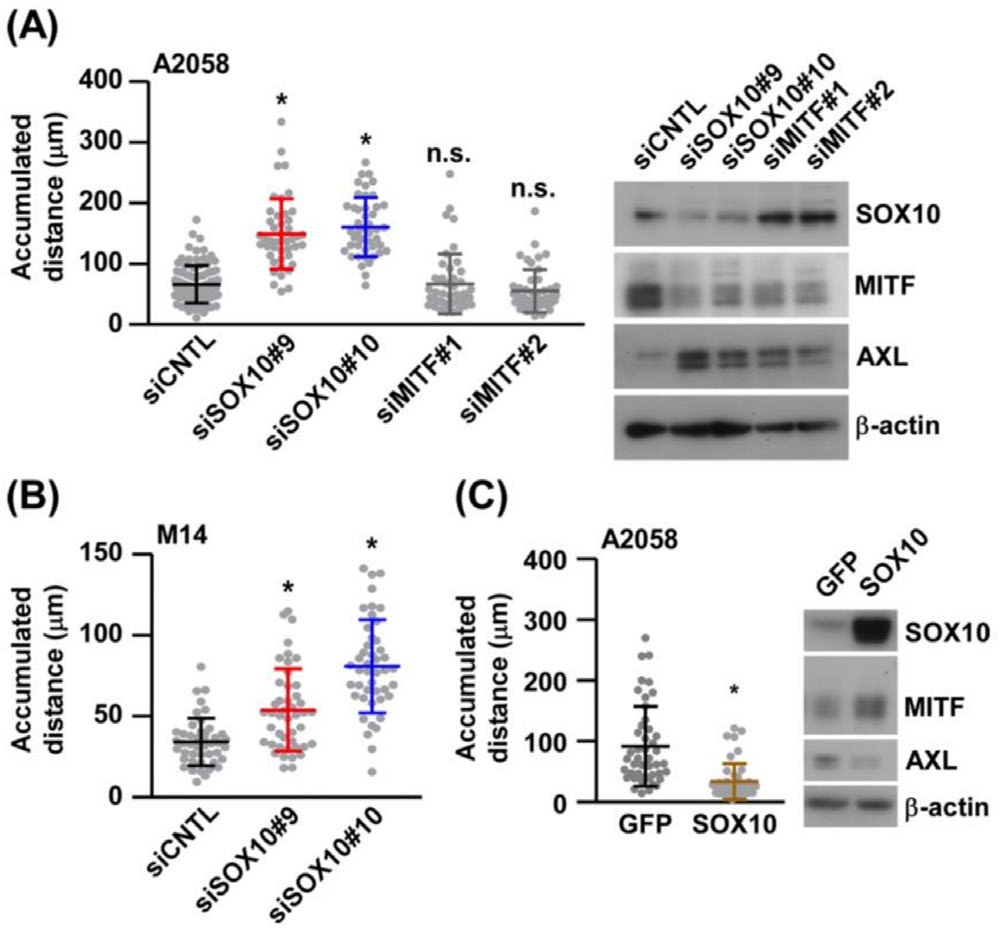

Melanoma is an aggressive form of skin cancer that can spread quickly to other parts of the body. While several genes have been associated with its growth, the precise molecular mechanisms that allow melanoma cells to migrate and form new tumors remain unclear.

Melanoma cells are known to exist in two main states: a proliferative state, in which they grow rapidly, and an invasive state, in which they acquire the ability to move and spread. The gene *SRY‐box 10* (SOX10) plays an important role in maintaining the proliferative state by helping in the development of melanocytes (pigment‐producing cells).

In this issue, Kaminaka et al. tried to understand how *SOX10* regulates melanoma cell movement and metastasis. They observed that reducing *SOX10* activity makes melanoma cells more mobile and aggressive, thereby promoting their spread.

Further experiments indicated that the loss of SOX10 increases the levels of two key proteins, *ITGA3* and *EphA2*, which help cancer cells attach to surrounding tissues and move independently. This change is controlled by another molecule, IRF1, which acts as a molecular switch linking *SOX10* to *ITGA3* and *EphA2*. Together, these molecules form a signaling chain—the IRF1–ITGA3/EphA2–FAK pathway—that enhances the ability of melanoma cells to metastasize.

Importantly, blocking IRF1 activity or inhibiting FAK, a protein that promotes cell migration, significantly reduced melanoma metastasis in laboratory models. This finding suggests that drugs targeting FAK, such as defactinib, could help prevent or slow melanoma spread in patients with low SOX10 activity.

Overall, these findings indicate that the loss of *SOX10* acts as a molecular switch that triggers melanoma cells to spread through the IRF1–ITGA3/EphA2–FAK pathway. Targeting this pathway, particularly through FAK inhibition, could represent a promising therapeutic strategy for controlling melanoma metastasis.

https://onlinelibrary.wiley.com/doi/full/10.1111/cas.70173

